# Pre‐embedded cervical circular stapled anastomosis in esophagectomy

**DOI:** 10.1111/1759-7714.13324

**Published:** 2020-02-04

**Authors:** Jie Li, Bin Wang, Tao Liang, Nan‐nan Guo, Ming Zhao

**Affiliations:** ^1^ Department of Thoracic Surgery First Medical Center of Chinese PLA General Hospital Beijing China; ^2^ Department of Cardiothoracic Surgery Fourth Medical Center of Chinese PLA General Hospital Beijing China

**Keywords:** Cervical anastomosis, esophagectomy, pre‐embedding, pre‐embedded

## Abstract

**Background:**

Mechanical anastomosis is now widely used in surgery for esophageal cancer. An anastomotic fistula is still the most dangerous complication in mechanical anastomosis, especially for patients who undergo cervical anastomosis. However, due to the high anastomosis position and limited space, conventional embedding and suspension are rarely performed. This study aimed to introduce the steps of an improved embedded method in cervical circular stapled anastomosis and evaluate its efficacy in reducing complications.

**Methods:**

In total, 31 patients who underwent minimally invasive esophagectomy were enrolled into the study. Pre‐embedded cervical esophagogastrostomy with a circular stapler was adopted after thoracoscopic and laparoscopic esophagectomy for esophageal cancer.

**Results:**

The results of surgical duration, blood loss, mean duration of hospitalization and operation complications such as anastomotic fistula, anastomotic stenosis and gastroesophageal reflux were recorded. The operative procedure lasted between 205–300 minutes with an average of 260.3 minutes. The postoperative recovery was good, with no complications such as anastomotic fistula, anastomotic stricture and pulmonary complication, except for two cases of gastroesophageal reflux. The postoperative hospital stay was 8–14 days with an average of 10.3 days.

**Conclusion:**

Our data revealed that pre‐embedded cervical circular stapled anastomosis is an alternative for patients with good stomach length, which can decrease the occurrence rate of anastomotic fistula by full peripheral embedding of anastomotic stoma.

**Key points:**

1. This new technique can significantly reduce the risk of anastomotic leakage.
2. This study adds further details enabling a smooth pre‐embedded procedure to be performed.

## Introduction

At present, surgical resection remains one of the most common treatments for esophageal cancer. However, the surgical procedure for esophageal cancer is complicated, and postoperative complications frequently occur, leading to high mortality rates. Esophagogastrostomy is currently the most typical approach for surgical anastomosis in esophagectomy.^1^ However, postoperative complications such as anastomotic fistula, anastomotic stricture and gastroesophageal reflux still exist,^2^, ^3^ which greatly affect the postoperative quality of patients' lives and in some cases are even life‐threatening.^4^ It has been reported that the incidence of cervical anastomotic fistula is about 10%–30%. The causes of postoperative anastomotic fistula are very complicated, among which the anastomotic method is an important factor affecting anastomotic healing and the main factor of anastomotic fistula. Therefore, anastomosis techniques used in esophageal surgery are always carefully considered by thoracic surgeons.^5^, ^6^

Currently, mechanical anastomosis is widely used in gastrointestinal tract anastomosis. Compared with hand‐sewn anastomosis, mechanical anastomosis has been accepted as a simple operation technique with low incidence of postoperative anastomotic fistula and stenosis.^5^ However, anastomotic fistula is still the most dangerous complication of mechanical anastomosis, especially in patients undergoing cervical anastomosis.^7^ In clinical practice, due to the high position of anastomosis, poor blood supply, high tension and limited operating space, common methods such as embedding and suspension are very difficult to apply in cervical anastomosis.^8^, ^9^

We herein introduce an improved embedded cervical esophagogastrostomy method using a circular stapler, and evaluate its efficacy in reducing the incidence of anastomotic fistula, stricture, and reflux.

## Methods

### Study design and participants

This study was approved by the Ethics Committee of Chinese PLA General Hospital. A total of 31 patients between January 2018 to January 2019 with esophageal cancer were enrolled into the study. All participants had signed their written informed consent.

### Preoperative assessment and preparation

Gastroscopic examination was performed preoperatively, and patients were pathologically confirmed to have esophageal cancer. After the evaluation showed no surgical contraindications, a modified cervical circular stapled anastomosis was adopted which was performed after thoracoscopic and laparoscopic esophagectomy of esophageal cancer and cervical esophagogastrostomy.

### Anastomosis procedure

**Figure 1:**
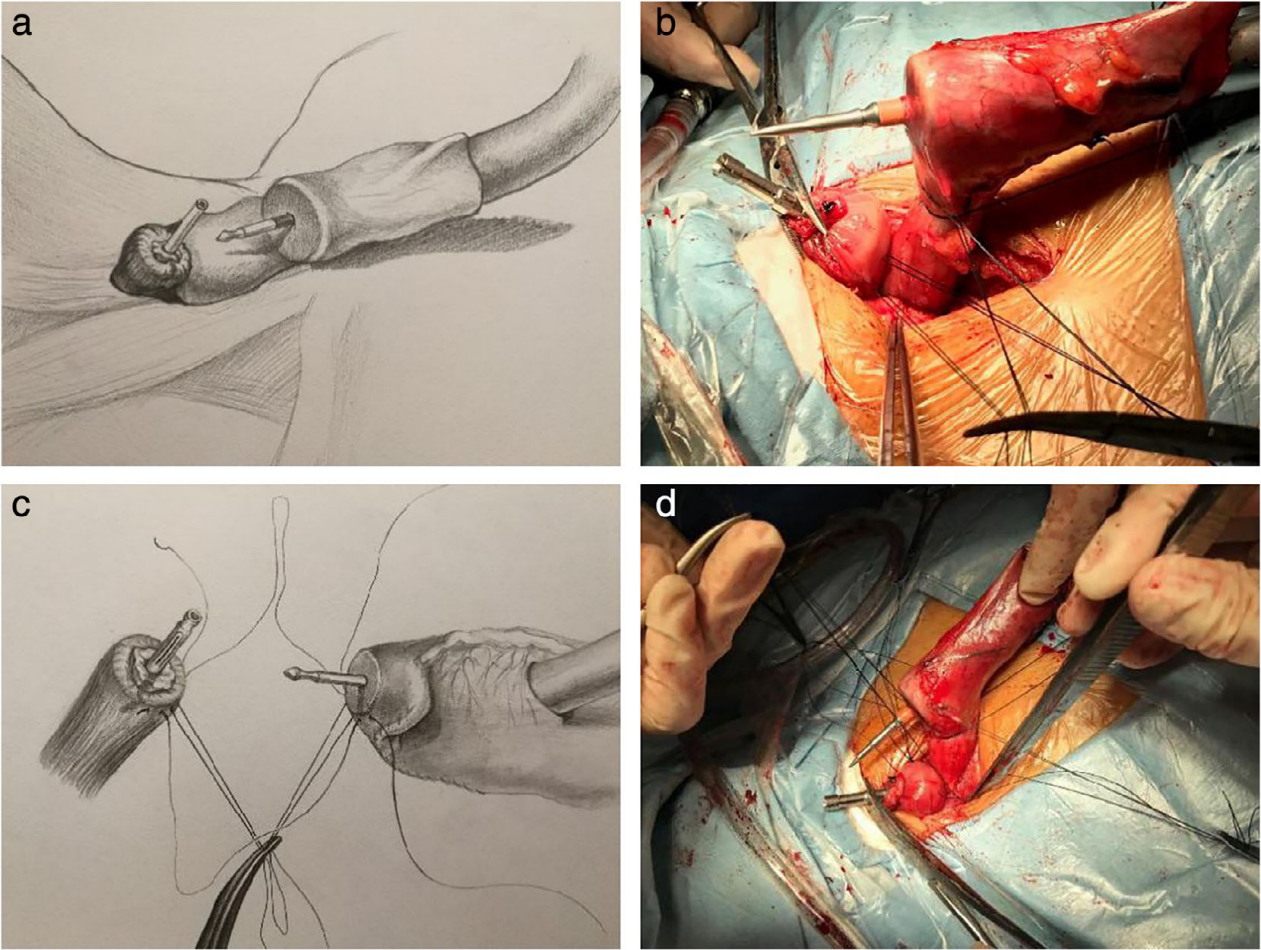
Demonstrates the instrument used for pre‐embedded cervical anastomosis. (**a**) The stapler puncture head was passed from the tubular stomach. (**b**) Pre‐embedded suture of the posterior wall of the tubular stomach and posterior wall of esophagus (two stitches on the right side). (**c**) Pre‐embedded suture of the posterior wall of the tubular stomach and posterior wall of the esophagus (third stitch). (**d**) Pre‐embedded suture of the posterior wall of the tubular stomach and posterior wall of the esophagus (fourth stitch).

After splitting the cervical esophagus, a purse‐string suture was performed at the proximal end of the esophageal stump. The anvil of the stapler was then inserted into the stump lumen and ligated for later use. The stapler was placed in a stoma at the end of the tubular stomach. After the puncture head was passed from the tubular stomach wall, sutures were made respectively on the posterior wall of tubular stomach seromuscular layer approximately 1 cm surrounding the anastomosis and the corresponding posterior esophageal wall muscular layer with four stitches, and threads were retained (Fig 1a,b). Anastomosis was performed after docking the stapler with the anvil, and the stapler was then removed carefully to ensure that none of threads had been cut by the stapler. Finally, the four stitches were knotted in turn. The anterior wall of the anastomotic stoma was sutured and embedded in the same way with a total of 8–10 stitches to achieve the effect of full peripheral embedding (Fig 1c,d). After stapling the gastric stoma, absorbable suture threads were used to perform whole‐layer suture for reinforcement plus seromuscular layer embedding. A drainage tube was retained after flushing the cervical anastomotic stoma.

### Postoperative treatment

Anti‐infection, nutritional support, and other treatments were routinely given postoperatively. The cervical drainage tube was removed after 24 hours of postoperative observation without bleeding. Drinking was attempted on the fifth to the seventh day after surgery, and stepwise transitional diet was advanced after confirmation that no adverse reaction had occurred.

### Follow‐up and outcomes

Patients were followed‐up for up to three months after discharge, and all patients underwent barium meal examination to evaluate the integrity of the anastomosis. The primary outcome was the incidence of anastomotic fistula within 30 days after surgery to evaluate the safety and efficacy of the new technique in reducing anastomotic fistula. The secondary outcomes were the overall morbidity and mortality at 30 days after surgery.

### Variables and statistical analysis

Basic demographic data of 31 patients were recorded including sex, age, pathological stage, tumor location, type of postoperative complication (anastomotic fistula, anastomotic stenosis, anastomotic reflux and pulmonary complication), surgical duration, blood loss and duration of hospitalization. Data were analyzed with SPSS software. Data were mean ± SD, or N (%). SD: Standard deviation.

## Results

### Patient characteristics

A total of 31 patients with esophageal cancer were enrolled into the study of which 26 were men and five were women, with a median age of 60 years (59.0 ± 6.78 years). The tumor position was 21–35 cm from the incisors. All patients underwent thoracoscopic and laparoscopic esophagectomy of esophageal cancer, followed by cervical esophagogastrostomy (Table 1).

**Table 1:** Patient demographics (*n* = 31)

Index	Patients, *n* (%)
Age (year)	59.0 ± 6.78
Sex	
Female	26 (84%)
Male	5 (16%)
Pathological stage	
I	14 (45%)
II	7 (23%)
III	10 (32%)
Tumor location	
Upper	7 (23%)
Middle	17 (54%)
Lower	7 (23%)

Data are Mean ± SD, or N (%). SD, Standard deviation.

### Postoperative mortality and morbidity

There was no morbidity during the study period. In addition, the average lengths of the operation time and postoperative hospital stay were 260.3 minutes (260.3 ± 22.5 minutes) and 10.3 days (8–14 days), respectively. After three months of follow‐up, the postoperative recovery was good, and swallowing function had not been significantly affected postoperatively. No complications such as anastomotic fistula, anastomotic stricture and pulmonary complication had occurred, except for two cases of gastroesophageal reflux (Table 2). Both patients were advised to eat little and take 20 mg omeprazole enteric‐coated tablets orally once a day until the symptoms improved.

**Table 2:** Postoperative surgical outcomes

Index	Patients, *n* (%)
Anastomotic fistula	0
Anastomotic stenosis	0
Gastroesophageal reflux	2 (6%)
Pulmonary complication	0
Surgical duration (min)	260.3 ± 22.5
Blood loss (mL)	66.1 ± 46.6
Mean duration of hospitalization (days, range)	10.3 (range = 8–14)

Data are mean ± SD, or N (%). SD, Standard deviation.

## Discussion

Esophageal cancer can be treated by various surgical procedures, the most common of which is gastric transposition for esophageal replacement and esophagogastrostomy. At present, common esophagogastrostomy methods include hand‐sewn and mechanical anastomosis.^10^, ^11^ Manual full‐thickness anastomosis is the traditional esophagogastrostomy approach. However, because of its long operative duration, the incidence of anastomotic fistula is high and it is less commonly used nowadays.^12^

Mechanical anastomosis mainly consists of circular stapled anastomosis and linear stapled anastomosis (including delta‐shaped anastomosis).^13^ Compared with hand‐sewn anastomosis, linear stapled anastomosis can greatly reduce the incidence of anastomotic fistula and anastomotic stricture.^14^, ^15^ However, this type of anastomosis requires the esophageal stump to be dissociated long enough to enable the insertion of the linear stapler, which is difficult to achieve in patients with a short neck or higher tumor location when performing cervical anastomosis. Additionally, the anastomotic stoma made by linear stapled anastomosis is too large, which can easily cause gastroesophageal reflux.^16^ What is more, patients may experience discomfort when swallowing after a cervical anastomosis due to an excessive residual end of the esophageal stump.^17^ On the contrary, the esophageal stump does not need to be dissociated as long when performing a circular stapled anastomosis. In addition, since a circular stapled anastomosis is closer to normal digestive tract structure, the incidence of gastroesophageal reflux is significantly lower than that of linear anastomosis, without indication of swallowing discomfort; however, the incidence of anastomotic fistula is not significantly different.^18^ Similarly, in their study, Honda *et al*. demonstrated that there was a shorter operative period for circular stapled anastomoses procedures, but that it did not diminish anastomotic leakage and could even augment the rate of stenosis.^5^

To the best of our knowledge, high anastomotic position, increased gastric tension, and poor blood supply in cervical anastomosis may contribute to the enhanced incidence of anastomotic fistula.^19^, ^20^ Embedding of the anastomotic stoma and stomach suspension are common measures to ameliorate tension at the anastomotic stoma during intrathoracic anastomosis, which can effectively decrease the occurrence of anastomotic fistula.^8^ However, due to the limited operating space in the neck and the abundant surrounding vessels and nerves, conventional anastomotic stoma suspension cannot be performed. Most conventional embedding can only be performed on the anterior wall of anastomotic stoma, while embedding the posterior wall of the anastomotic stoma is exceedingly difficult. Therefore, the posterior wall of the anastomotic stoma becomes a weak area, resulting in the occurrence of anastomotic fistula.^19^

Based on the cervical circular stapled anastomosis, the pre‐embedded method was used in this study to achieve full peripheral embedding of anastomotic stoma, which could effectively abate the tension at the anastomotic stoma. This method can greatly reduce the tension of the anastomotic stoma and effectively reduce the incidence of anastomotic fistula. This method is suitable for almost all neck anastomotic patients if the demand to dissociate the esophageal stump is not so high and is easy to perform. In order to prevent an anastomotic fistula, three key steps should be emphasized. First, the stomach tube should be extensively dissociated. Second, it is better to make the anastomotic stoma closer to the gastric arch. Third, a full peripheral embedding can be completed using the pre‐embedding skills.

In conclusion, the effect of the pre‐embedding method on the prevention of postoperative complications after cervical esophagogastrostomy was significant. Compared with traditional embedding, it has significant effects in reducing gastroesophageal reflux and eliminating anastomotic fistula, anastomotic stenosis and pulmonary complications, thereby improving the postoperative experience of patients with esophageal cancer. The main limitation of this research was the small sample size studied in a single center. In summary, pre‐embedded cervical circular stapled anastomosis can be an alternative for patients with good stomach length.

## Disclosure

There are no conflicts among the authors to be disclosed.
